# Acute Hyperkalaemia During General Anaesthesia in Two Cows

**DOI:** 10.3390/ani16213409

**Published:** 2026-10-29

**Authors:** Cristina Sánchez, Dany Elzahaby, Olivier Louis Levionnois

**Affiliations:** 1 Anaesthesiology Service, Department of Animal Medicine and Surgery, Veterinary Teaching Hospital, Veterinary Faculty, Complutense University of Madrid, Avda. Puerta de Hierro s/n, 28040 Madrid, Spain; csancv@hotmail.com; 2 School of Veterinary Medicine, Murdoch University, Perth 6150, Australia; dany.elzahaby@murdoch.edu.au; 3 Anaesthesiology and Pain Therapy Section, Department of Clinical Veterinary Medicine, Vetsuisse Faculty, University of Bern, 3012 Bern, Switzerland

**Keywords:** hyperkalaemia, potassium, ruminant, electrolyte imbalance, bradycardia

## Abstract

An 8-year-old, 600 kg, female Simmental cow and a 6-year-old, 662 kg, pregnant Hérens cow were referred for surgical repair of mandibular symphyseal fractures following trauma in two unrelated events. Pre-anaesthetic blood tests revealed mild hypokalaemia in the Simmental cow and mild hypocalcaemia in the Hérens cow. Anaesthesia was induced with xylazine, ketamine, and lidocaine, and maintained with isoflurane combined with a ketamine constant rate infusion. Throughout most of the procedures, physiological parameters remained stable, although mild hypercapnia and progressive bradycardia were noted. During surgical closure, both cows developed sudden severe bradycardia, followed by ventricular escape beats and short periods of asystole. Arterial blood gas analysis revealed acute severe hyperkalaemia, with potassium concentrations of 6.8 mmol/L in the Simmental cow and 8.66 mmol/L in the Hérens cow. Immediate treatment with a calcium–magnesium–glucose solution was initiated, resulting in rapid improvement in heart rate, restoration of sinus rhythm, and reduction in potassium concentrations. Both cows recovered uneventfully, with normal electrolyte values over the following 72 h. This report describes acute intra-anaesthetic hyperkalaemia in two cattle, including a pregnant animal, occurring without an identifiable predisposing factor. Continuous monitoring and rapid recognition of changes in cardiovascular status and electrolyte concentrations allowed prompt treatment and restoration of normal cardiac rhythm.

## Simple Summary

General anaesthesia is essential for many surgical procedures in cattle and is usually safe when animals are carefully monitored. However, rare complications can occur unexpectedly and may rapidly become life-threatening. This report describes two cows that developed a sudden increase in blood potassium concentration during surgery. Potassium is an important mineral that helps the heart beat normally, but excessive amounts can cause severe slowing of the heart and even temporary cardiac arrest. Pre-anaesthetic blood tests revealed only minor deviations from the reference intervals in potassium concentration in one cow and calcium concentration in the other, and no definitive cause for the subsequent hyperkalaemia was identified. The problem was recognised after unexpected changes in heart rhythm developed during surgery and was successfully treated with intravenous medication, allowing both cows to recover without further complications. Because this condition has rarely been reported in cattle, these cases increase awareness of an uncommon but serious anaesthetic complication. They also emphasise the importance of continuous monitoring during prolonged anaesthesia, as well as prompt diagnosis and treatment to maintain animal welfare and patient safety.

## 1. Introduction

Hyperkalaemia is a potentially life-threatening peri-anaesthetic complication as it may precipitate severe cardiac arrhythmias and cardiac arrest [1]. An increase in extracellular potassium concentration reduces the transmembrane resting potential, resulting in impaired myocardial excitability and conduction [2]. Characteristic electrocardiographic changes include alterations in P-wave morphology, T-wave configuration, QRS complex widening, and PR segment abnormalities, which may ultimately progress to asystole [3]. Reported causes of intraoperative hyperkalaemia include renal disorders (e.g., ureteral obstruction and uroperitoneum), transfusion of stored red blood cell concentrates, chronic heparin therapy, iatrogenic potassium chloride supplementation, Addisonian crisis, and has been linked to certain anaesthetic agents (medetomidine, propofol) [2,4].

Intra-anaesthetic hyperkalaemia has been recognised in both human and veterinary medicine. In veterinary species, it has been most frequently reported in dogs, horses (in which it may be associated with a genetic predisposition), large felids, and anecdotally in cats and cattle [4,5,6]. To the authors’ knowledge, only one bovine case has previously been published, involving a calf that developed severe hyperkalaemia during general anaesthesia without the typical electrocardiographic abnormalities associated with this electrolyte disturbance [6].

This report describes two cows that developed severe intraoperative hyperkalaemia. In both, hyperkalaemia was recognised following the sudden onset of severe bradycardia and transient episodes of asystole and resolved after prompt medical treatment. These cases highlight the importance of considering hyperkalaemia as a differential diagnosis in cattle that develop unexpected bradyarrhythmias during general anaesthesia.

## 2. Case Presentation

The first case involved an 8-year-old Simmental cow, weighing approximately 600 kg, that was referred to our farm animal teaching hospital following trauma to the head and hip overnight (approximately 12 h ago). The second case involved a 6-year-old Hérens cow, weighing 662 kg, that was referred following facial trauma and associated swelling for several days (unclear duration). On presentation, both cows appeared otherwise healthy; general physical examination findings, including hydration status, were unremarkable, apart from the evident facial injuries and subsequent pain. The second cow (Hérens) was 6 months and 10 days pregnant at the time of presentation. Radiographic examination of the mandible in both cows revealed a mandibular symphyseal fracture, requiring surgical repair under general anaesthesia. The two cases were managed approximately 1 year apart. Pre-anaesthetic blood parameters collected on the day of surgery showed only minor deviations from local reference ranges (Table 1), with mild hypokalaemia in the Simmental cow and mild hypocalcaemia in the Hérens cow.

The Simmental cow (Case 1) underwent emergency anaesthesia on the day of presentation, whereas the Hèrens cow (Case 2) was fasted from feed for 24 h prior to anaesthesia, with water available ad libitum. Both were administered flunixin 2.2 mg/kg (Vetaflumex Neo; Provet AG, Lyssach, Switzerland) and benzylpenicillin sodium 30.000 IU/kg (Penicillin Natrium Streuli; Streuli Pharma, Switzerland) intravenously prior to anaesthesia. Owing to pregnancy, the Hèrens cow also received isoxsuprin 0.3 mg/kg (20 mL) (Degraspasmin; Graeub AG, Bern, Switzerland) intramuscularly. Both cows were premedicated with xylazine 0.2 mg/kg (Xylazin Streuli; Streuli Pharma, Uznach, Switzerland) intramuscularly. After ten minutes, the patients were adequately sedated, and anaesthesia was induced with ketamine 5 mg/kg (Ketanarkon, Streuli Pharma, Uznach, Switzerland) and lidocaine 1 mg/kg (Lidocaine 2%, Streuli Pharma, Uznach, Switzerland) intravenously. The trachea was intubated, and anaesthesia was maintained with isoflurane in oxygen, titrated to clinical effect. In both cases, a constant rate infusion of ketamine was administered at 1 mg/kg/h intravenously, and lactated Ringer’s solution at approximately 5 mL/kg/h. Volume-controlled mechanical ventilation was promptly initiated after intubation, using a tidal volume of 6–7 L and a respiratory rate of 8 breaths/min, resulting in a peak inspiratory pressure of 18–24 cmH_2_O. Ventilator settings were subsequently adjusted as required to maintain adequate oxygenation and normocapnia. Both cows were positioned in left lateral recumbency for the procedure.

Perioperative management was similar in both cows. Physiological parameters including heart rate (HR), respiratory rate (ƒR), arterial oxygen saturation (SpO_2_), capnography, end-tidal carbon dioxide tension (EtCO_2_), fraction of inspired oxygen (FiO_2_), end-tidal isoflurane concentration, and oesophageal temperature were recorded at 5 min intervals. A 20 G catheter was placed in the right auricular artery and connected to a pressure transducer (via a saline-filled column) positioned and zeroed at the level of the right atrium. The catheter was used for continuous invasive arterial blood pressure monitoring and intermittent arterial blood sampling for blood gas analysis. Bilateral mental nerve blocks were performed using a palpation-guided technique with lidocaine (Lidocaine 2%, Streuli Pharma, Switzerland), administering 5 mL per site, as previously described in buffalo and horses [7,8]. Prior to start of surgery, butorphanol was administered intravenously at 0.05 mg/kg (Morphasol; Graeub). For both cows, surgery commenced approximately 45–50 min after tracheal intubation. Throughout the procedures, the patients’ vital parameters remained within normal limits with mild variability. Both cases experienced initial mild hypercapnia (EtCO_2_ 48–53 mmHg) and tachycardia (100–120 bpm), normalising progressively during the course of the anaesthetic (heart rate at 70–80 bpm).

In the Simmental cow (Case 1), marked bradycardia developed abruptly 115 min after induction of anaesthesia, with heart rate decreasing from approximately 70 to 20–25 beats/min. This was accompanied by widened QRS complexes, irregular ventricular beats and intermittent periods of asystole. Invasive blood pressure decreased after the arrhythmia started, while normocapnia was maintained. In the Hèrens cow (Case 2), a rapid decrease in heart rate was observed after 200 min of uneventful anaesthesia, falling from 60 to 20 beats/min within less than one minute and this was followed by occasional ventricular escape beats characterized by widened QRS complexes, lacking P-wave precursors, and intermittent periods of asystole lasting approximately 7 s. Electrocardiographic changes occurred simultaneously with acute reductions in EtCO_2_, invasive arterial pressure, and plethysmograph waveforms.

In both cases, the arrhythmias developed while the surgical procedure was ending (skin closure) more than 30 min after discontinuation of the ketamine constant rate infusion. Isoflurane administration was discontinued immediately, and an arterial blood sample was collected for blood gas analysis (RAPIDPoint 500 e blood gas analyzer, Siemens Healthcare Diagnostics Inc., Norwood, MA, USA). Arterial sampling (3 mL) was performed anaerobically in a purpose-designed, dry-heparinized blood-gas syringe at room temperature, after discarding the first 3 mL, removing air and gently mixing, and immediately analyzing as whole blood. No sign of haemolysis was noticed. Arterial blood gas analysis revealed severe hyperkalaemia with a potassium concentration of 6.8 mmol/L in the Simmental cow and 8.66 mmol/L in the Hèrens cow. At the time hyperkalaemia was detected, arterial blood pH was 7.44 in the Simmental cow and 7.36 in the Hérens cow. Neither cow presented with acidemia, but in the Hérens cow, PaCO_2_ was mildly increased (47.3 mmHg; bovine arterial reference interval, 35–44 mmHg), suggesting a possible respiratory acidifying component despite the absence of acidemia. Further characterization of the acid–base status was not possible because additional parameters, including bicarbonate concentration and base excess, were unavailable.

Treatment of hyperkalaemia was initiated immediately while both cows were still on the surgical table. In the first cow (Simmental), two intravenous doses of atropine (0.01 mg/kg) and one dose of adrenaline (0.005 mg/kg) were administered to treat severe bradyarrhythmia and hypotension; however, these interventions failed to produce sustained clinical improvement. In the second cow (Hérens), atropine was considered but ultimately not administered because the bradyarrhythmia and sinus pauses were intermittent and short-lived. Specific treatment for hyperkalaemia was then initiated, consisting of intravenous administration of calcium salts to stabilize the cardiac membrane and glucose to promote intracellular potassium redistribution. A solution consisting of calcium, magnesium, and glucose monohydrate (Gluca-Magnesium; Biokema SA, Bussigny, Switzerland) was administered. Each cow received 1 L of the solution, equivalent to approximately 160–180 mg/kg of glucose, 22–25 mg/kg of calcium and 15–17 mg/kg of magnesium. The infusion was started immediately after the decision to initiate specific treatment and completed over approximately 20 min; however, the exact infusion duration was not recorded.

Clinical improvement was observed within approximately 5 min of commencing the infusion. In both cows, heart rate increased to approximately 70 beats/min, with resolution of the bradyarrhythmia and fewer sinus arrest episodes.

Surgery was quickly ceased, and both cows were transferred to the recovery area. Isotonic saline (NaCl 0.9%) infusion was initiated at 5 mL/kg/h. A repeat electrolyte analysis performed 20 min after treatment commenced revealed a decrease in blood potassium from 6.8 to 5.47 mmol/L in the Simmental cow and from 8.66 to 6.97 mmol/L in the Hèrens cow. Concurrent electrocardiographic monitoring showed marked improvement with restoration of sinus rhythm and heart rates of 120–140 beats/min. Both cows recovered softly, were extubated after the return of the swallow reflex approximately 10–15 min after discontinuation of isoflurane, and moved into sternal recumbency approximately 15–20 min later. During the subsequent 72-h postoperative monitoring period, serial electrolyte measurements remained within the local reference intervals. The total duration of anaesthesia was 130 min in the first case and 210 min in the second case.

## 3. Discussion

This report describes two cases of intraoperative hyperkalaemia in cows undergoing general anaesthesia for mandibular symphyseal fracture repair. Hyperkalaemia is a potentially life-threatening electrolyte disturbance that can lead to significant cardiovascular consequences, including bradyarrhythmias, conduction blocks, and cardiac arrest.

While it is well-recognised in small animal anaesthesia and human medicine, hyperkalaemia is rarely reported in cattle under general anaesthesia. To date, only a single bovine case has been published: a calf undergoing anaesthesia for umbilical fistula resection, in which unexpected intraoperative hyperkalaemia developed without associated electrocardiographic changes at diagnosis, although arterial potassium rose acutely above normal limits (7.60 mmol/L) [6]. In contrast, the present cases exhibited marked electrocardiographic disturbances, progressive bradycardia culminating in sinus arrest and transient asystole, coinciding with potassium levels in the severe range (6.8 and 8.66 mmol/L). These findings are consistent with the progressive electrocardiographic manifestations of hyperkalaemia described in ruminants, including attenuation and eventual disappearance of P waves, widening of the QRS complex, and prominent peaked T waves, although the relationship between blood potassium concentration and ECG abnormalities is not absolute [9].

Several mechanisms may contribute to intraoperative hyperkalaemia in ruminants. In the calf described by Gonzalez Gomez et al [6], intraoperative hyperkalaemia was detected in association with respiratory acidosis (PaCO_2_ 66 mmHg), likely resulting from spontaneous ventilation under general anaesthesia. Mechanical ventilation was subsequently initiated to correct the hypercapnia and acid–base imbalance because acidosis may have contributed to intracellular–extracellular potassium shifts, as hydrogen ions move into cells in exchange for potassium [10,11]. In contrast, the cows in the present report developed mild hypercapnia (EtCO_2_ 48–53 mmHg) without acidemia, so a pharmacological approach combining calcium, magnesium and glucose was required to restore cardiac rhythm and reduce blood potassium rapidly.

Other potential predisposing factors must be considered. Prolonged recumbency and trauma may have contributed to muscle injury, potentially promoting the release of intracellular potassium into the extracellular compartment. Muscle damage and rhabdomyolysis are recognized potential causes of hyperkalaemia in veterinary patients [12,13]. However, the extent of muscle injury was not established in either cow, and their contribution to the observed hyperkalaemia remains speculative. Pregnancy-related physiological changes were also considered; however, there is currently insufficient evidence to establish pregnancy itself as a predisposing factor for hyperkalaemia in cattle [14]. Moreover, xylazine, a commonly used α2-adrenergic agonist in cattle, has been associated with transient hyperkalaemia, likely due to suppression of insulin secretion and altered potassium redistribution between intracellular and extracellular compartments [15,16]. In addition, xylazine-induced hyperglycaemia, whether after bolus administration or continuous infusion, may further exacerbate hyperkalaemia through osmotic cellular dehydration and impaired potassium uptake in the context of insulin deficiency [16,17,18]. Non-steroidal anti-inflammatory drugs (NSAIDs) can also promote hyperkalaemia by suppressing prostaglandin-mediated renin release, resulting in hypoaldosteronism [19]. This effect has been described with ketorolac but not with flunixin, which was used in this case, and appears to occur more frequently in patients with pre-existing renal compromise [20]. While these pharmacological factors may contribute to the development of electrolyte disturbances, they are part of routine standard anaesthetic care and are unlikely to have triggered hyperkalaemia alone.

The duration of anaesthesia has also been proposed as a risk factor for intraoperative hyperkalaemia [21]. In dogs, a retrospective study reported that all affected patients had procedures lasting longer than 60 min [4]. Similarly, McEntire et al. (2020) identified anaesthetic duration as a negative predictor in large felids, observing a direct correlation between anaesthetic time and blood potassium concentrations [22]. In the present cases, the timing of hyperkalaemia onset cannot be determined, as blood potassium concentrations were not measured serially throughout anaesthesia. However, electrocardiographic changes that prompted the first measurement of elevated potassium concentrations occurred after 115 and 200 min of anaesthesia in the two cases. In the previously reported calf, hyperkalaemia was detected at 120 min post-induction [6]. These findings highlight that clinically relevant hyperkalaemia may develop during prolonged anaesthesia in cattle, although the temporal pattern of potassium accumulation remains unclear.

Treatment in these cases consisted of intravenous administration of calcium salts to stabilise the myocardium, combined with glucose infusion to promote intracellular potassium translocation. Intravenous calcium raises the threshold potential, restoring the gradient between resting and threshold potentials and thereby re-establishing membrane excitability [23]. Its effect is rapid, occurring within minutes. Glucose infusion, with or without insulin, is commonly used to lower extracellular potassium; however, the use of insulin in food-producing animals is not permitted under European Union legislation (Regulation (EU) No. 37/2010). For this reason, treatment in the present cases was restricted to glucose infusion and isotonic crystalloids, fortunately resulting in successful clinical improvement.

Adrenaline administration in one cow was indicated to restore cardiac output during severe hyperkalaemia. Besides its positive chronotropic and inotropic effects mediated through β_1_-adrenergic stimulation, adrenaline may also facilitate intracellular potassium uptake through β_2_-receptor activation and stimulation of Na^+^/K^+^-ATPase activity, thereby contributing to the reduction in potassium concentration [24]. Although its potassium-lowering effect is well established in human medicine, evidence in veterinary species remains scarce [25]. Conversely, abrupt withdrawal of adrenaline infusion has been associated with rebound hyperkalaemia and recurrence of life-threatening arrhythmias in human patients, highlighting the importance of careful dose titration during catecholamine therapy [24].

Given that the Hèrens cow was in late gestation, the potential impact of maternal anaesthesia and intraoperative complications on foetal well-being must also be considered. Drugs such as xylazine and ketamine have been associated with increased uterine tone and alterations in uteroplacental blood flow, which could increase the risk of preterm labour or foetal compromise [26]. In addition, foetal asphyxia may result from maternal hypoxemia, reduced uteroplacental perfusion, or impaired maternal-foetal gas exchange. Similarly, uteroplacental circulation is not autoregulated and therefore depends directly on maternal blood pressure and cardiac output; if maternal hypotension is not corrected, foetal oxygenation can be rapidly compromised [27]. In this case, cardiovascular instability was transient, and maternal oxygenation was maintained through mechanical ventilation. However, as follow-up data were unavailable, the subsequent course of the pregnancy and the potential impact on the foetus could not be assessed.

## 4. Conclusions

This report highlights the importance of anticipating and promptly recognising hyperkalaemia during general anaesthesia in cattle, as rapid diagnosis and intervention can be lifesaving. Although uncommon, clinicians should remain vigilant when prolonged anaesthetic times, pharmacological factors, or metabolic alterations are present, since these may predispose patients to this complication. The occurrence in both a Simmental cow and a pregnant Hérens cow suggests that this problem may not be limited to a single breed or physiological status. Further reports are warranted to better characterise risk factors and to establish preventive and therapeutic strategies in cattle and other large animal species.

## Figures and Tables

**Table 1:** Pre-anaesthetic blood parameters were generally within the reference intervals (presented within parenthesis), except for a mildly decreased potassium concentration in the Simmental cow.

Parameter	Simmental	Hérens
Hematocrit (%)	26 (24–35)	29 (24–40)
Leucocytes (×10^9^/L)	4.12 (4.0–10.0)	N/A
Total solid (g/L)	65.8 (64–82)	N/A
Na (mmol/L)	141 (133–145)	136 (132–146)
K (mmol/L)	3.71 * (3.9–5.75)	3.95 (3.9–5.8)
Cl (mmol/L)	101 (91–110)	103 (95–110)
Ca (mmol/L)	2.24 (1.94–2.82)	1.09 ** (1.05–1.30)

N/A: not available. Reference intervals correspond to those provided by the analyser manufacturer. * Out of reference interval. ** ionized Ca.

## Data Availability

The original contributions presented in this case are included in the article. Further inquiries can be directed to the corresponding author.

## Abbreviations

The following abbreviations are used in this manuscript:

HR	Hear rate
ƒR	Respiratory rate
SpO_2_	Peripheral oxygen saturation
EtCO_2_	End-tidal carbon dioxide
FiO_2_	Fraction of inspired oxygen
